# The magnitude and associated factors of immediate postpartum anemia among women who gave birth in Ethiopia: systematic review and meta-analysis, 2023

**DOI:** 10.1186/s12884-024-06495-y

**Published:** 2024-04-25

**Authors:** Aysheshim Asnake Abneh, Tadele Derbew Kassie, Sintayehu Shiferaw Gelaw

**Affiliations:** Department of public health, College of Medicine and Health Science, Debre Markos University, Debre Markos, Ethiopia

**Keywords:** Anemia, Immediate postpartum, Magnitude, Associated factors, Ethiopia, Systematic review, meta-analysis

## Abstract

**Background:**

The immediate postpartum period is a very crucial phase for both the life of the mother and her newborn baby. Anemia is the most indirect leading cause of maternal mortality. However, anemia in the immediate postpartum period is a neglected public health problem in Ethiopia. Therefore, this systematic review and meta-analysis aimed to estimate the pooled magnitude of immediate postpartum anemia and the pooled effect size of associated factors in Ethiopia.

**Methods:**

Searching of published studies done through PubMed, Medline, Cochrane, African index Medicus, List of Reference Index, Hinari, and Google Scholar. This systematic review and meta-analysis follow the Preferred Reporting Items for Systematic Review and Meta-Analysis (PRISMA) godliness. The quality of studies was assessed by using a Newcastle- Ottawa Scale (NOS) assessment tool. Analysis was performed using a random effect model by using STATA 17 version software. Egger’s weighted regression and I^2^ test were used to evaluate publication bias and heterogeneity respectively.

**Results:**

In this systematic review and meta-analysis, a total of 6 studies were included. The pooled magnitude of immediate postpartum anemia in Ethiopia was 27% (95%CI: 22, 32). Instrumental mode of delivery (OR = 3.14, 95%CI: 2.03, 4.24), mid-upper arm circumference (MUAC) measurement < 23 cm (OR = 3.19, 95%CI: 1.35, 5.03), Antepartum Hemorrhage (OR = 4.75, 95%CI: 2.46, 7.03), postpartum hemorrhage (OR = 4.67, 95%CI: 2.80, 6.55), and no iron/foliate supplementation (OR = 2.72, 95%CI: 1.85, 3.60) were the identified factors associated with developing anemia in the immediate postpartum period.

**Conclusion:**

The overall pooled magnitude of anemia in the immediate postpartum period among Ethiopian women was still a moderate public health problem. Instrumental mode of delivery, mid upper arm circumference (MUAC) measurement < 23 cm, antepartum hemorrhage, postpartum hemorrhage, and no iron/foliate supplementation were the identified factors associated with higher odds of developing anemia among immediate postpartum women in Ethiopia. Therefore, midwives, and doctors, shall focus on prevention of maternal hemorrhage, nutritional advice and counseling including iron /foliate supplementation, and avoid unnecessary instrumental delivery to prevent and reduce anemia related maternal mortality and morbidity in Ethiopia.

**PROSPERO registration:**

CRD42023437414 with registration date on 02/08/2023.

**Supplementary Information:**

The online version contains supplementary material available at 10.1186/s12884-024-06495-y.

## Introduction

### Background

The postpartum period is the most crucial phase for both the life of the mother and her newborn babies. Even though a large proportion of maternal and neonatal deaths occur during the first two days after delivery, this is the most neglected period for the provision of quality care especially in low-resource setting countries including Ethiopia [1, 2]. Anemia is a condition in which the number of red blood cells or hemoglobin concentration within them is lower than the normal cut-off values, for this reason impairing the blood’s ability to transport oxygen to meet physiological needs. It is an indicator of both poor nutrition, and poor health, having a significant consequences for the women and their children [3, 4]. Even though there is no consensus on the definition of postpartum anemia, it can be inferred from the definitions provided by different scholars depending on the duration of the postpartum period. Thus it can be defined as hemoglobin levels less than 10 g/dl, less than 11 g/dl, and less than 12 g/dl cut-off values within the first 48 h after delivery, at one week, and six weeks postpartum period respectively [5–7]. Similarly, immediate postpartum anemia can be defined as hemoglobin level less than the cut-off values of 10 g/dl within the first 48 h after a woman gave childbirth [8–10]. Even though progress has been made decreasing in maternal mortality and morbidity in many countries in the world; there is still evidence of no change or a continuous increment in the indirect causes of maternal mortality [11–13]. Globally around 35% of all maternal deaths are attributable to indirect causes of maternal mortality; of which around 7% are due to anemia and anemia also contributed to 2.3% of maternal deaths among all causes [14, 15]. The direct and indirect contribution of anemia to maternal death or near- miss has been demonstrated in countries with low resource setting [16, 17] In addition to this, a severe form of postpartum anemia increases maternal death, and postpartum infection by threefold during the postpartum period [18, 19]. Another evidence revealed that a severe form of anemia in the postpartum period increases maternal death by twofold compared with non-anemic women [20]. Globally an estimated 500,000 maternal deaths occurred annually related to pregnancy, labor, and delivery; of which around 20% were due to postpartum anemia [21, 22]. In our country Ethiopia, postpartum anemia is one of the leading indirect causes of maternal mortality. As evidenced by the Ethiopian Demography and Health Survey (EDHS 2016) postpartum anemia contributed to maternal mortality [23]. Anemia in the immediate postpartum period is also strongly associated with poor quality of life, increased maternal infection, fatigue, reduced cognitive ability, and postpartum depression later on. The outcome of these may in turn affect or slow down the infant’s development [24–26]. The magnitude of immediate postpartum anemia particularly two days after childbirth was relatively low in the developed world and varies from 10 to 30%: However, in low and middle-income countries it was as high as from 50–80% [24]. Varies of studies were conducted across the world regarding with immediate postpartum anemia. A study done in Madrid Spain to determine anemia among women after childbirth shows that the overall magnitude of immediate postpartum anemia on the second day after delivery was 29%, of which 5% were classified as severe anemia [9]. Similar studies conducted in Germany to determine early postpartum anemia shows 22% [27], Kasturba Medical College in India 26.5% [28], and Pakistan 47% [29] of women were anemic.

Another study done in China revealed that the overall incidence of anemia among immediate postpartum women was 57% [30]. Sub-Saharan Africa and Southeast Asia have one of the highest prevalence of anemia in the immediate postpartum period. A study conducted in Nigeria to determine the magnitude of immediate postpartum anemia shows that the overall magnitude of anemia immediately after 48 h of delivery was 46% [25]. In Ethiopia contradicting the anemia reduction plan 2020; the magnitude of postpartum anemia among postnatal women increased from 18% in 2011 to 28% in 2016 [23, 31]. The Ethiopian government launched an anemia reduction plan (below 12% by the end of 2025) and different strategies to reduce anemia among reproductive-age women while it is still a public health problem and unlikely to achieve the national targets [32]. This is due to poor health service utilization, low socio-economic status of women, low adherence to iron and folic acid supplementation during pregnancy, and blood loss due to bleeding during childbirth [33, 34].

The studies conducted around the world revealed that there are so many multiple and interlinked factors identified like poor quality health care services, poverty, low iron supplementation, inadequate micronutrient intake, nutritional deficiencies, and high infectious disease in developing countries attributed to the presence of higher rates of immediate postpartum anemia compared with developed countries [35–37]. Moreover, factors like maternal age, low educational status of the mother, rural residence, ante-natal care follow-up (ANC) [38], cesarean mode of delivery, anemia during pregnancy, antepartum hemorrhage (APH), postpartum hemorrhage (PPH) and malaria infection were factors associated with the immediate postpartum anemia [25, 29, 39–41].

In Ethiopia, some studies are conducted to assess the magnitude and determinant factors of immediate postpartum anemia among women during the immediate postpartum period [42–44]. However, these separate studies reported the magnitude of immediate postpartum anemia among immediate postpartum women in Ethiopia ranged from 21.6% study conducted at the east Gojam zone [42] to 41.4% study conducted at Shewarobit health facilities, North Shewa. These showed that there was considerable variation and uncertainty related to the magnitude of immediate postpartum anemia and its associated factors among women who gave birth across the nation. Therefore, our study aimed to determine the pooled magnitude and associated factors of immediate postpartum anemia among women who give birth in Ethiopia to provide evidence-based information for policymakers and stakeholders to design and implement evidence-based interventions to avert anemia morbidity and associated mortality among immediate postpartum women in Ethiopia.

### Objective of the review

This systematic review and meta-analysis had two main objectives to be addressed by the study. These were:

1. To determine the estimated pooled magnitude of immediate postpartum anemia among women who gave birth in Ethiopia.
2. To identify the estimated pooled effect sizes of factors associated with immediate postpartum anemia among women who gave birth in Ethiopia.

## Methods

### Study design

Systematic review and meta-analysis.

#### Study selection and eligibility criteria

In this review, studies that were conducted only in Ethiopia with any observational study designs (cross-sectional, case control, and cohort) regardless of the publication status (both published and unpublished) were considered as eligible, and included in this systematic review and meta-analysis. Other eligibility criteria were full text articles conducted in English language, and reported the magnitude and associated factors of immediate postpartum anemia were included. However, conference abstracts, editorials, case reports, review articles, articles without full-text availability (since the quality assessment of articles are not possible without full text), qualitative studies were excluded from this study. In this review, studies were screened using eligibility criteria by three authors independently and finally cross-checked for consistency. Furthermore, any disparities between authors were solved through further discussion, and consensus.

#### Study participants

In this systematic review and meta-analysis, the study participants were immediate postpartum women who gave birth in Ethiopia.

#### Types of studies

Types of studies to be included in this, systematic review and meta-analysis were all observational studies (cross-sectional, case control, and cohort). However, unfortunately there was no study found other than cross-sectional study design in the setting area, due to this reason only studies done by cross-sectional stud design were included in this study.

### Measurements of the outcome

This systematic review and meta-analysis had two main outcomes. The first outcome had to estimate the pooled magnitude of immediate postpartum anemia among women who gave birth in Ethiopia. As defined by various scholars’ immediate postpartum anemia is a condition in which a hemoglobin concentration is below 10 g/dl within the first 48 h after a woman gave childbirth [9, 10, 45]. The magnitude of immediate postpartum anemia was calculated by dividing the total number of immediate postpartum women who had anemia by the total number of study participants, and then multiplied by 100. The second outcome was the association factors. For the second outcome, the pooled odds ratio with 95%CI was used to measure the levels of association between immediate postpartum anemia and factors.

### Searching strategy

The review of all published and unpublished (Gray literature) studies was done by using the following major databases; MEDLINE, PubMed, Hinari, Cochrane Library, African Index Medicus, and other sources like use of List of Reference Index, and Google scholar to retrieve new articles. Endnote X7 software was utilized to retrieve and organize the studies identified through the search strategy, as well as to eliminate any duplicate records. In order to locate relevant studies within the search databases, the following search terms were employed: (magnitude) OR (prevalence) OR (proportion) OR (incidence) AND (associated factors) OR (risk factors) OR (predictors) OR (determinants) AND (immediate postpartum) OR (immediate postnatal) OR (early postpartum) AND (anemia) OR (anaemia) OR (low hemoglobin) OR (iron deficiency) AND (women who give birth) OR (women who are postnatal period) AND (Ethiopia). Theses search terms were used individually as well as in combination, utilizing Boolean operators like “OR” and “AND”. The last search was done on December 20, 2023. When conducting this review, the Preferred Reporting Items for Systematic Reviews and Meta-Analysis (PRISMA) guideline was strictly followed.

### Data extraction processes

Firstly, we developed the data extraction format agreed upon by all authors by using Microsoft Excel version 16 to extract the necessary data from the selected articles. The developed data extraction format has the following structure: (1) Author detail which contains name and year of publication, (2) study year, (3) study design, (4) study setting (health facility versus community based), (5) sample size, (6) study population, (7) sampling procedures, (8) data collection procedures, (9) region where the study was done, (10) specific area where the study was done, and 11) the response rate. In addition to these, the number of immediate postpartum women who had anemia, along with 95% confidence interval was included in the prepared data extraction format for the first outcome (magnitude of immediate postpartum anemia). For the second outcome (factors associated with immediate postpartum anemia), data were collected in the form of two by two table, and the log odds ratio for each factors was calculated based on the primary study findings. Secondly, the data were extracted independently by three authors and checked for the consistency of the extracted data. When inconsistency occurred, the studies were reviewed for the second time. Finally, disagreements were resolved by verification and further discussion.

### Quality assessment

The quality of the primary studies was assessed by using the Newcastle Ottawa Scale (NOS) assessment tool adapted for cross-sectional studies [46]. The assessment tool has a total of 7 elements such as representativeness of the cases, sample size, response rate, ascertainment of the exposure, controlling of confounders, assessment of the outcome, and statistical tests. The first four elements were category I (the selection component), and scores a maximum of 5 points, the fifth one was category II (comparability) and score a maximum of 2 points, and the last two elements were the category III (outcome components) and scores a maximum of 3 points. Based on this, it was interpreted as a score of: 0–4 as poor, 5–6 as fair, 7–8 as good, and 9–10 as very good. A score of 7 and above were considered as high quality (eligible for the study), and enrolled in this systematic review and meta-analysis (Table 1). This was done by the three authors independently, and when any disparities occurred, review done for the second time. If still there were any disparities, then the differences were solved by further discussion and consensus.

**Table 1:** Quality assessment of the included studies by using the Newcastle Ottawa Assessment (NOS) tool adapted for cross-sectional study in Ethiopia, 2023

First author & reference					Comparability	Outcome			
	Representativeness of sample size	Sample size justified	Non- Response rate	Ascertainment of the exposure (max **)	Cofounding controlled max(**)	Outcome assessment (max **)	Statistics	Total Score (max 10*)	Overall quality
Abebaw et al.	*	*	*	*	**	**	*	9*	Good
Gizaw et al.	*	*	*	*	**	**	*	9*	Good
Girma et al.	-	-	*	*	**	**	*	7*	Good
Agmassie et al.	*	*	*	*	*	**	*	8*	Good
Nigussie	*	-	*	*	*	**	*	7*	Good
Bireda et al.	*	*	*	*	**	**	*	9*	Good

*Note*: * refers to a score of point one, and ** refers to a score of point two

### Data synthesis and analysis

The extracted data in the Microsoft Excel format was exported to Stata version 17 statistical software for analysis. Since we considered heterogeneity among studies, a random effect meta-analysis model with a restricted maximum likelihood method was used to compute the estimated pooled magnitude of immediate postpartum anemia. In this systematic review and meta-analysis, the Forest plot was used to show the pooled estimate with a 95% confidence interval (95% CI). Statistical heterogeneity was evaluated by using the values of I-squared (I^2)^ test. The I^2^ statistic represents the percentage that can be attributed to variability between studies. The values of I^2^ statistic varies between 0 and 100%, and the heterogeneity of the included studies was interpreted as follows: low heterogeneity for < 50%, moderate heterogeneity for 50–75%, and high heterogeneity for greater than or equal to 75% [47]. The pooled effect sizes of factors associated with immediate postpartum anemia were estimated as an odds ratio. At the final stage P values less than 0.05 were considered statistically significant for all analyses.

## Results

### Search results

**Fig. 1:**
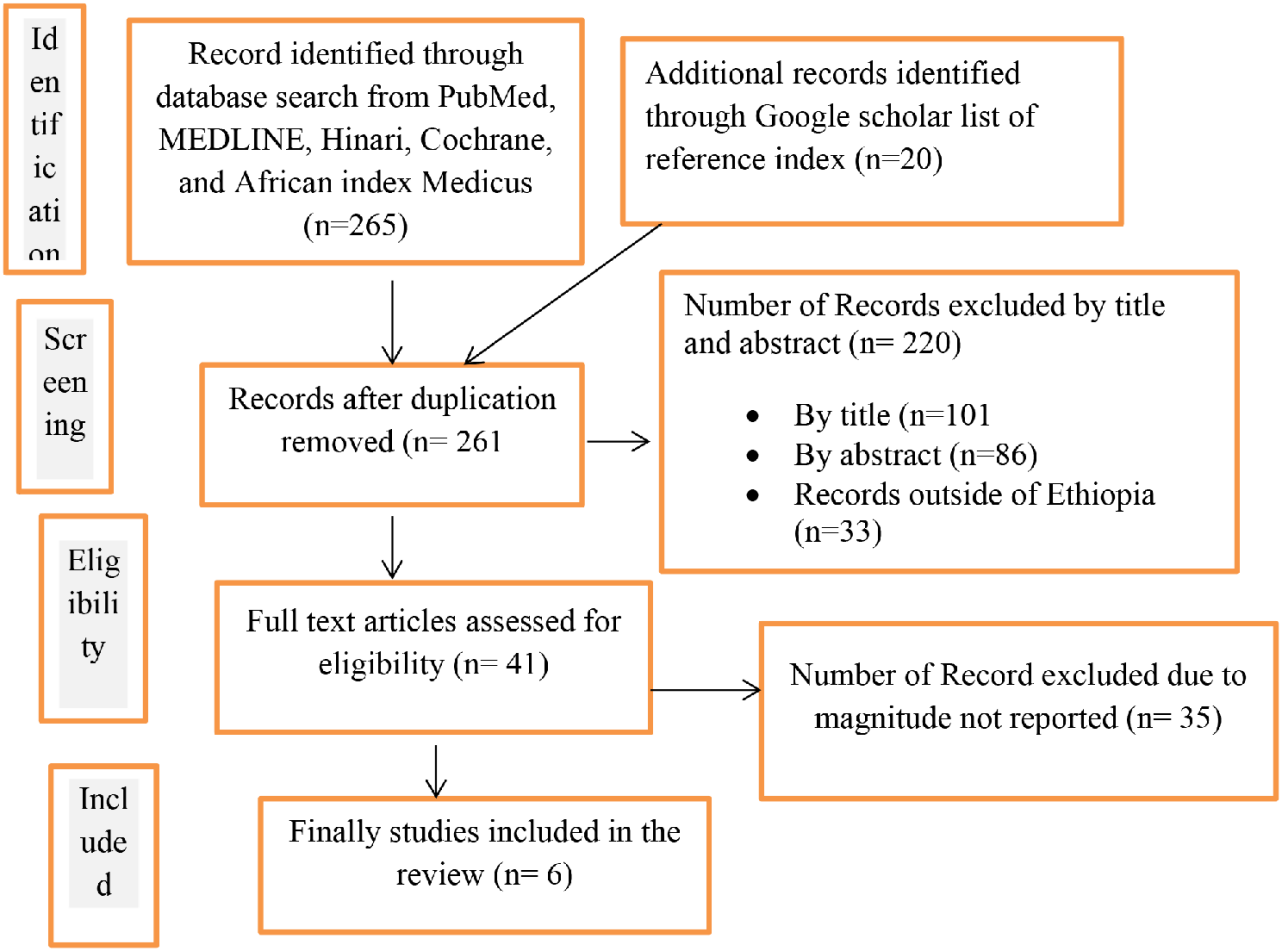
Show the data selection and eligibility of the magnitude of immediate postpartum anemia among women who gave birth in Ethiopia, 2023

In this systematic review and meta-analysis, a total of 285 records from Google Scholar, PubMed, MENDLINE, African Index Medicus, Cochrane Library, List of Reference Index, and Hinari were searched. Of these around 24 studies were excluded due to duplication. After assessing the titles and their abstracts we also excluded 220 study records because these articles were not related to our review. After assessing 41 full articles, 35 were further excluded due to the specific outcome variable was not reported (the magnitude not reported). Even though, unpublished studies (Gray literature were searched through Google scholar, no study was found. Finally, six studies were included in this systematic review and meta-analysis (see Fig. 1).

### Characteristics of the included studies

This systematic review and meta-analysis employed 6 primary studies with a total of 2,394 study participants. Among the 6 studies, 3 studies were conducted in the Amhara region, one study was conducted at Dire-Dawa city administration, one study was conducted in Harari regional state and another study was conducted in the Tigray region. All studies were cross-sectional in study design, and conducted in a health facility based. In this systematic review and meta-analysis, the sample size ranges from 236 conducted at Mekele hospital in Tigray region to 484 reported from Harari regional stat. The lowest magnitude of immediate postpartum anemia was 21.6% (95%CI: 17.9, 25.3) conducted from East Gojjam, Amhara region and the highest magnitude of immediate postpartum anemia was 41.4% (95%CI: 35.9, 46.9) reported in Shewarobit, North Shewa, and this was also found in Amhara region. Regarding to the sampling technique they used, only one study (the study done at Mekele in Tigray region) was done by using consecutive sampling. The rest five studies were done by using systematic random sampling (Table 2).

**Table 2:** Summary of studies included in the systematic review and meta-analysis of the magnitude and associated factors of immediate postpartum anemia in Ethiopia, 2023

Authors	Publication year	Region	Sample sizes	Magnitude in% with 95%CI	Study design	Area	Study setting	Sampling technique
Abebaw et al [43]	2020	Amhara	424	24.3 (20.2, 28.4)	Cross-sectional	DMCSH	Facility based	Systematic random S
GT Abebe et al [44]	2022	Harari	484	28.1 (21.3, 34.8)	Cross-sectional	Harari	Facility based	Systematic RS
Girma [48]	2020	Tigray	236	24.2 (18.7, 29.6)	Cross-sectional	Mekele H	Facility based	consecutive
Agmassie et al. 2023 [42]	2023	Amhara	467	21.6 (17.9, 25.3)	Cross-sectional	East Gojjam	Facility based	Systematic RS
A Niguss	2022	Amhara	307	41.4 (35.9, 46.9)	Cross-sectional	Shewarobit	Facility based	Systematic RS
A Bireda [8]	2022	Dire Dawa	476	26.9 (22.9, 30.8)	Cross-sectional	Dire Dawa	Facility based	Systematic RS

Note: DMCSH refers to Debre Markos specialized hospital, H refers to hospital, and RS refers to random sampling

### Quality of the included studies

During our quality assessment, we found that all of the included studies exhibited reliable methodological quality. The Newcastle-Ottawa Scale 9NOS) scores ranged between 7 and 9 out of a total score of 10. This indicated that each primary study had high quality (Table 1).

### Magnitude of immediate postpartum anemia

**Fig. 2:**
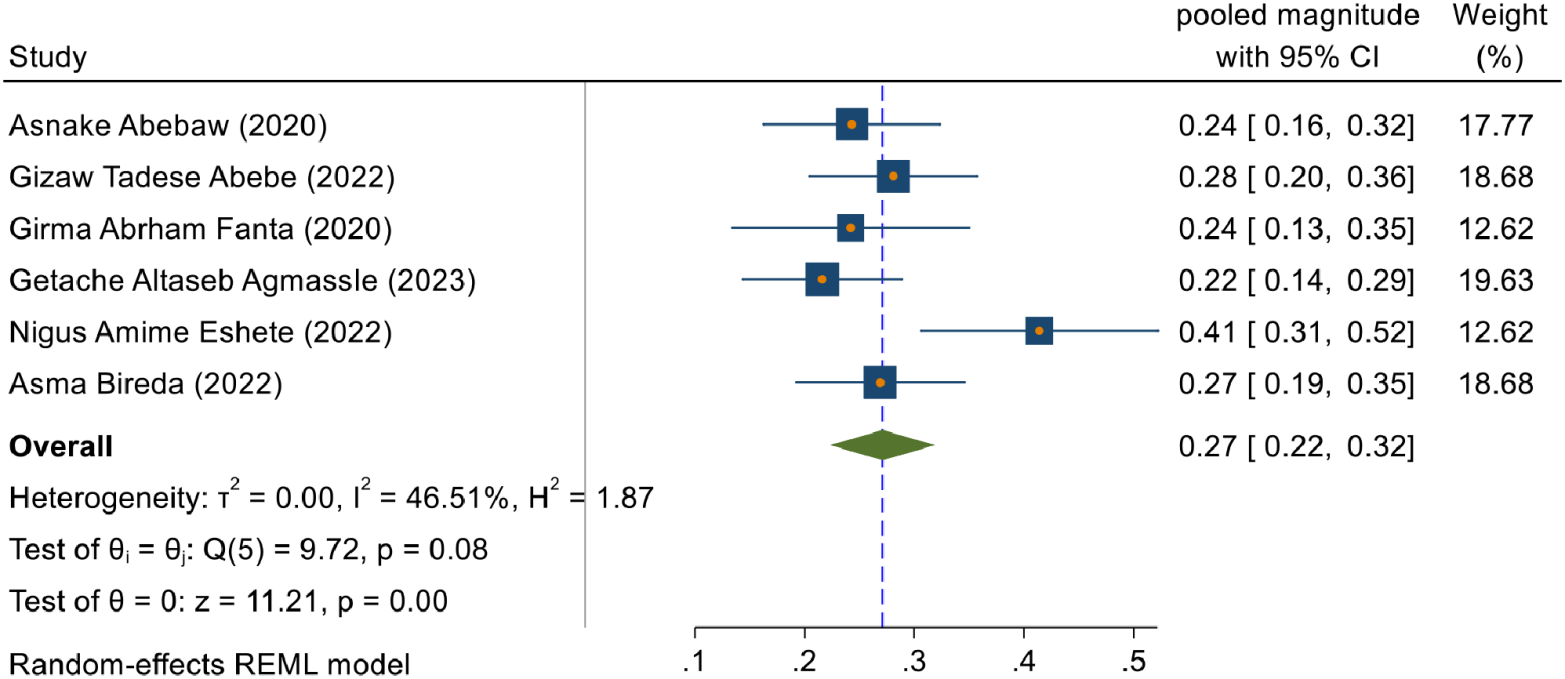
The estimated pooled magnitude of immediate postpartum anemia among women who gave birth in Ethiopia, 2023

A random effect meta-analysis model was used to estimate the pooled magnitude of immediate postpartum anemia. Accordingly, the pooled magnitude of immediate postpartum anemia in Ethiopia was 27% (95% CI: 22, 32) with a heterogeneity of (I^2^ = 46.51%) (Fig. 2).

### Heterogeneity and publication bias

**Fig. 3:**
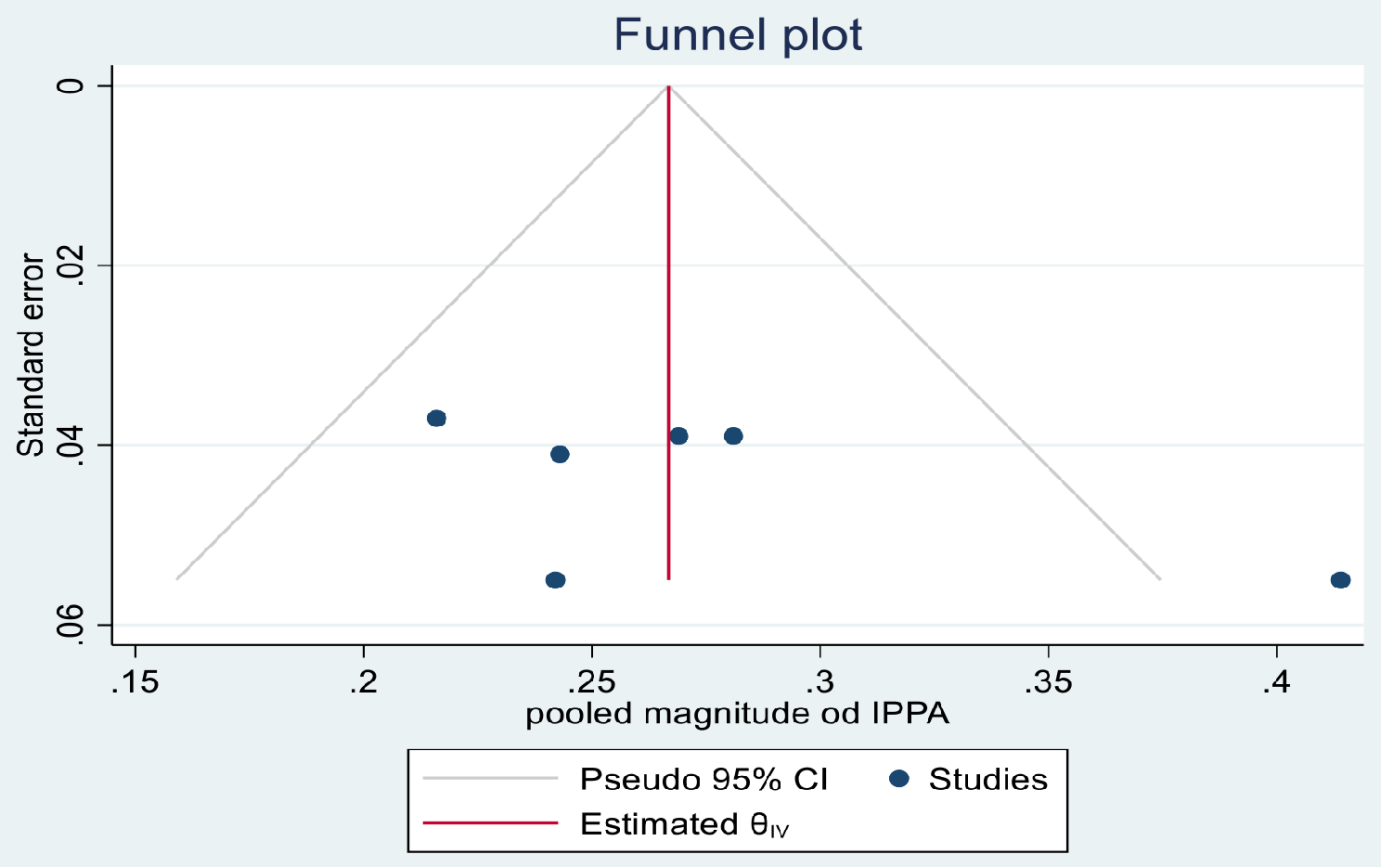
Funnel plot to test publication bias of magnitude of immediate postpartum anemia in Ethiopia, 2023

Heterogeneity of the studies was assessed by the value of I^2^ test. Accordingly, there was no significant heterogeneity among the included studies since the vale was 46.51%. Publication bias was also assessed by using funnel plot (Fig. 3). The graphical funnel plot showed the asymmetry of the studies, but the Egger’s regression test showed there was not significant publication bias.

### Factors associated with immediate postpartum anemia

In this systematic review and meta-analysis, factors associated with immediate postpartum anemia among women who give birth in Ethiopia were also identified. For factor analysis, a random effect model with a restricted maximum likelihood method was used to compute variables since we consider heterogeneity among the included studies. Those variables reported had a statistically significant association with the magnitude of immediate postpartum anemia in at least two primary studies were incorporated in the current meta-analysis. Accordingly, instrumental mode of delivery (either forceps or vacuum), mid upper arm circumference (MUAC) measurements of women < 23 cm, history of PPH during the current childbirth, history of APH during the most recent pregnancy, and iron-foliate supplementation, were significantly associated with immediate postpartum anemia (Table 3).

**Fig. 4:**
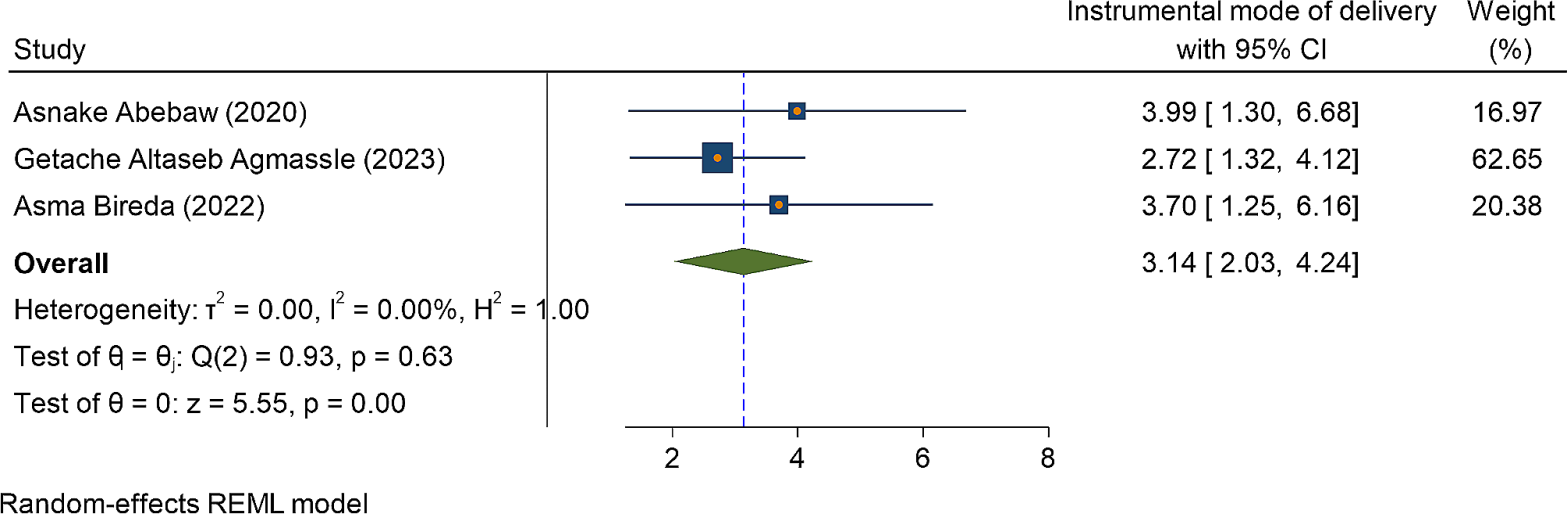
The association between instrumental mode of delivery (forceps or vacuum) and immediate postpartum anemia in Ethiopia, 2023

**Fig. 5:**
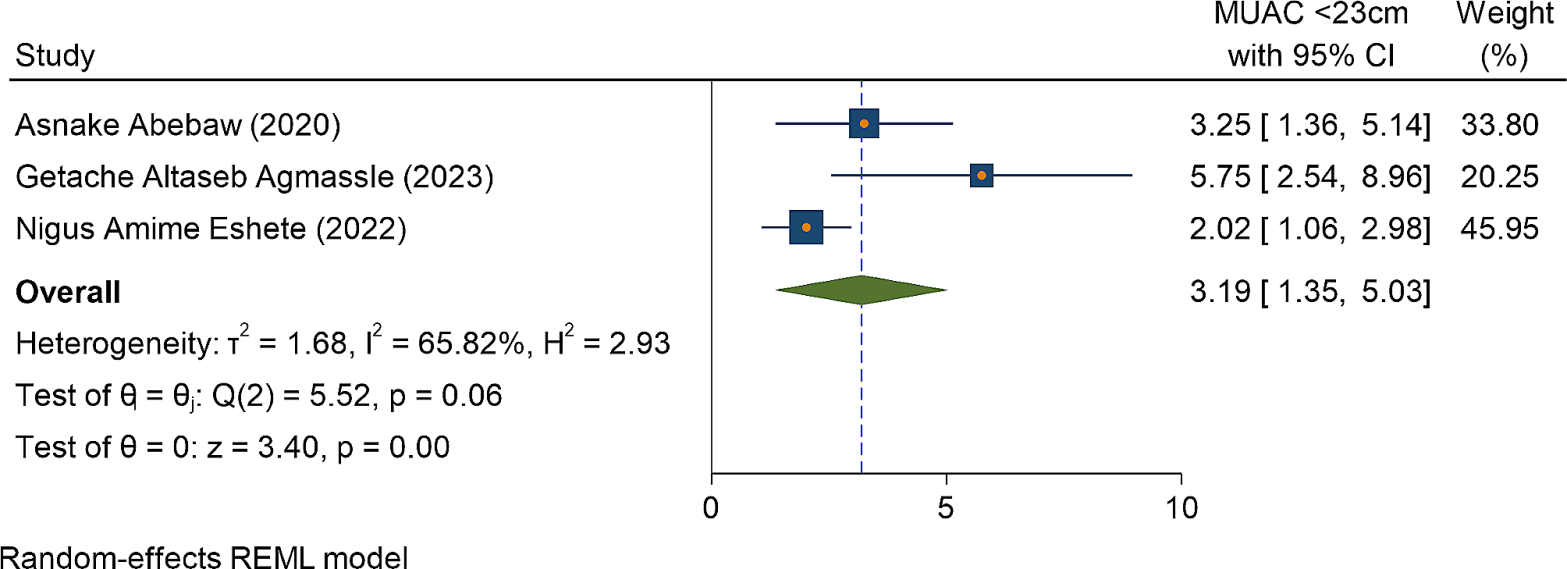
The association between measurement of MUAC < 23 cm and the magnitude of immediate postpartum anemia among immediate postpartum women in Ethiopia, 2023

The Instrumental mode of delivery (vacuum or forceps) is one of the independent predictors of immediate postpartum anemia among the two primary studies. Hence, women who gave birth with either vacuum or forceps delivery were three times more likely to develop anemia as compared to women who gave birth with spontaneous vaginal delivery OR = 3.14(95%CI: 2.03, 4.24) (Fig. 4). The nutritional status of women also affects women’s anemic status. Accordingly, among three primary studies, women whose MUAC measurement less than 23 cm were three times more likely to develop anemia as compared to their counterparts OR = 3.19 (95%ci: 1.35, 5.03) with heterogeneity of I^2^ = 65.8% (Fig. 5).

**Fig. 6:**
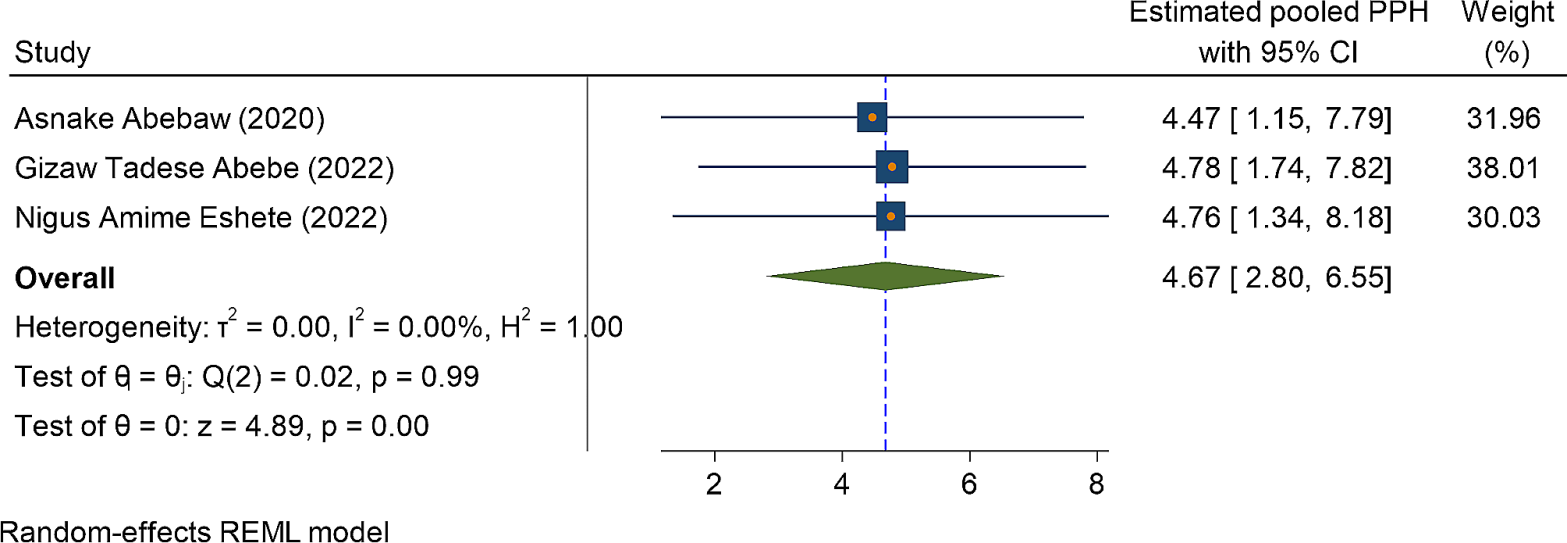
The association between the factor PPH and the magnitude of immediate postpartum anemia among immediate postpartum women in Ethiopia, 2023

**Fig. 7:**
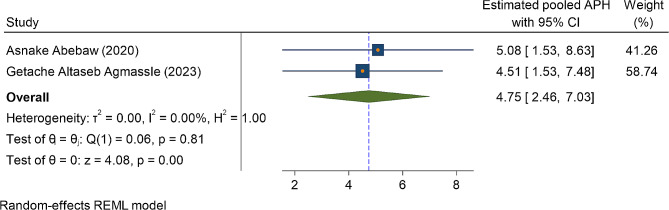
The association between the factor of APH and the magnitude of immediate postpartum anemia among immediate postpartum women in Ethiopia

**Fig. 8:**
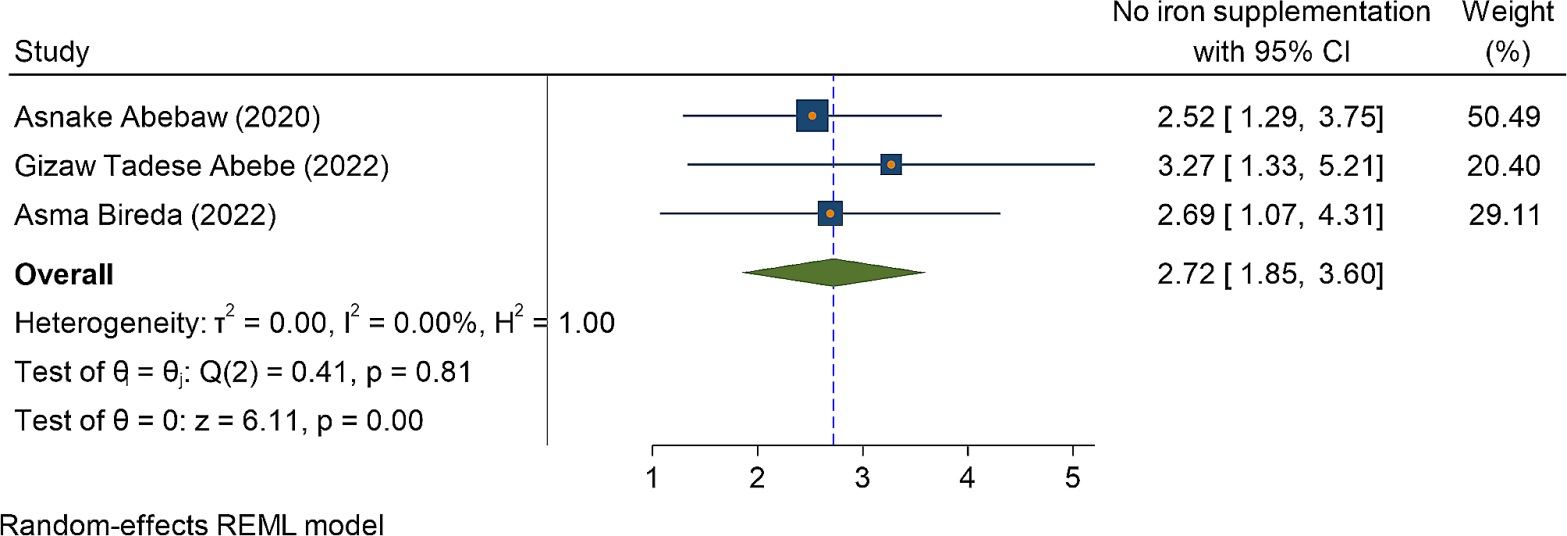
The association between the factor of no iron and folic acid supplementation, and the magnitude of immediate postpartum anemia among immediate postpartum women in Ethiopia, 2023

In three primary studies, a history of blood loss immediately after current childbirth (PPH) was significantly associated with anemia in the immediate postpartum period. Accordingly, the odds of postpartum anemia were higher among postpartum women who had history of developing postpartum hemorrhage (PPH) OR = 4.67(95%CI: 2.80, 6.55) (Fig. 6). Similarly, among two primary studies, women who had a history of antepartum hemorrhage in the most recent pregnancy (APH) were four and half times more likely to be anemic as compared to their counterparts OR = 4.75(95%CI: 2.46, 7.03) (Fig. 7). Lastly, iron and folic acid supplementation were one of the predictors of anemia in the immediate postpartum period. The odd of immediate PPA was higher among women who had no iron and folic acid supplementation during the most recent pregnancy as compared to their counterparts OR = 2.72(95%CI: 1.85, 3.60) (Fig. 8)).

**Table 3:** Summary of the associated factors of immediate postpartum anemia among women who gave birth in Ethiopia, 2023

Variables	Number of studies	Studies included	Pooled odds ratio with 95%CI
Instrumental mode of delivery	3	Asnake Abebew et al. (2020)	3.14 (2.03, 4.24)
		Getachew Altaseb et al. (2023)	
		Asma Bireda et al. (2022)	
MUAC measurement less than 23 cm	3	Asnake Abebew et al. (2020)	3.19(1.35, 5.03)
		Getachew Altaseb et al. (2023)	
		Nigus Amime et al. (2022)	
History of PPH	3	Gzachew Tadesse et al. (2022)	4.67 (2.80, 6.55)
		Asnake Abebew et al. (2020)	
		Nigus Amime et al. (2022)	
History of APH	2	Asnake Abebew et al. (2020)	4.75 (2.46, 7.03)
		Getachew Altaseb et al. (2023)	
No iron and folic acid supplementation	3	Asnake Abebew et al. (2020)	2.72 (1.85, 3.60)
		Gzachew Tadesse et al. (2022)	
		Asma Bireda et al. (2022)	

## Discussion

Even though, anemia in the immediate postpartum period is still a public health problem especially in low resource setting countries, there was no analogous meta- analysis study conducted in this specific research question within the area. This systematic review and meta-analysis were carried out to estimate the pooled magnitude and associated factors of immediate postpartum anemia among women who gave birth in Ethiopia. Accordingly, this systematic review and meta-analysis revealed that the estimated pooled magnitude of immediate postpartum anemia was 27% (95%CI: 22, 32). Despite of several meta-analysis studies were conducted regarding anemia during pregnancy and postpartum period across the world including the study settings, there was a lack of analogues meta-analysis study on the immediate postpartum anemia. This makes a difficult in the discussion part in this study.

This systematic review and meta- analysis also determine the pooled effect of factors associated with immediate postpartum anemia among mothers who gave childbirth. Accordingly, instrumental (vacuum or forceps) mode of delivery, nutritional status of women (MUAC < 23 cm), history of blood loose during pregnancy and at childbirth, and iron/foliate supplementation were significantly associated with immediate postpartum anemia among postnatal women in Ethiopia. The odds of immediate postpartum anemia were almost three times higher among women who gave birth either by vacuum or forceps as compared to women who gave birth with spontaneous vaginal delivery (SVD). This finding was supported by the study done [49], and the study done in Israel [50]. This is might be due to instrumental delivery (forceps or vacuum) increase risk of episiotomy, laceration on the premium, vaginal wall, cervix, and sometimes extended to uterus which increased risk of blood loss, and reduction of red blood cell RBC) [51]. Spontaneous lacerations of the cervix usually undiagnosed by most clinicians and left unrepaired until a women bleed a lot.

The odd of anemia in the immediate postpartum women were three times higher among women whose MUAC measurement less than 23 cm as compared to their counterparts. This is supported by the study done in Myanmar [52], and the study done at Dar es Salam, Tanzania [53]. This could be explained by anemia during pregnancy and in the postpartum period highly related to nutritional deficiencies (especially iron and folic acid). Evidences shows that MUAC measurement less than 23 cm indicates poor muscle mass, lack of adequate energy intake, poor iron store and low hemoglobin concentration [54, 55]. MUAC is one of the indicators of the nutritional status of women during pregnancy, and after giving childbirth. Poor nutrition and lack of attention predisposes a woman to iron deficiency anemia [56].

Another determinant factor of IPPA was blood lost either during pregnancy in the form of APH or after pregnancy at the time of giving childbirth (PPH). The odds of IPPA were increased by fivefold among women who had history of hemorrhage in the antepartum period as compared to women who didn’t have history of hemorrhage in the antepartum period. Similarly, the odds of IPPA were four and half times higher among women who experienced blood lost immediately after childbirth as compared to their counterparts. These findings were supported by the study done in Germany [27], and Saudi Arabia [45]. The possible explanation might be due to massive bleeding before childbirth during pregnancy because of placental separation, and placenta previa or during and after childbirth due to uterine atony or laceration on the birth canal leads to the depletion of iron store in the blood volume [57, 58]. Moreover, in every milliliter of blood lost, almost a half milligram of iron depleted in the blood volume [59, 60].

Lastly, taking the recommend dose of iron and folic acid during the most recent pregnancy affects the anemic status of women in the postpartum period. Hence, the odds of developing IPPA were two and half times higher among women who didn’t take iron and foliate supplementation during pregnancy as compared to postpartum women who took iron and folic acid during their pregnancy. This finding was supported by the study done. The possible explanation might be due to iron is a necessary replacement for physiological physical growth of the fetus during pregnancy, and blood lost during childbirth and in the postpartum period. Evidence show that consumption of the recommended 90 iron containing tablet supplement during pregnancy can reduce maternal anemia by at least 70% [61]. A multilevel analysis conducted in Ethiopia show that more that of 60% of reproductive age women took iron/folic acid supplementation during their recent pregnancy; but only 20% of pregnant women took iron for 90 pills or more [62], but the national report in 2019 was much lower than this, which was only 11% of pregnant women received 90 + iron tablets. This might be contributed to high prevalence of anemia in the immediate postpartum period [63]. Iron deficiency accounts for 75% of cases of non-physiologic anemia during pregnancy [64]. Moreover, iron deficiency is one of the most micronutrient deficiency which affects half of the world’s population [14, 65].

## Strength and limitations of the study

### Strength of the study

Since this study is a systematic review and meta-analysis, the findings provide a more conclusive result than any individual studies conducted in Ethiopia.

### Limitations of the study

This study has some limitations. The first thing is since all studies included in this systematic review and meta-analysis were cross-sectional study designs, the result cannot show the real cause-effect relationships between the immediate postpartum anemia and the identified associated factors. Secondly, Currently, Ethiopia has more than 12 regions, and these regions have geographical variation (low land and high land, high agricultural productive versus low agricultural productive, high versus low quality of maternal health care services). Since most of the studies included in this meta-analysis were from areas of high agricultural productivity, and high qualities of maternal health care services, the finding may be underestimated in Ethiopia.

## Conclusion

This systematic review and meta- analysis indicated that anemia in the immediate postpartum women in Ethiopian was still a moderate public health problem. Instrumental mode of delivery (either vacuum or forceps), maternal undernutrition measured by MUAC < 23 cm and history of blood lost either during pregnancy or after giving childbirth were factors associated with higher odds of developing anemia among women of immediate postpartum period in Ethiopia. Moreover, no or lack of nutritional supplementations such as iron and folic acid during pregnancy was another identified predictor of developing anemia among women in the immediate postpartum period in Ethiopia. Accordingly, the government of Ethiopia needs to monitor and evaluate the implementation and effeteness of nutritional programs in Ethiopia to strengthen comprehensive multi-sectorial and facility-based interventions such as food diversification, food fortification, and micro-nutrient supplementations, and education on the recommended iron/foliate supplementation to prevent and reduce anemia morbidity and mortality. In addition to this, midwives, doctors, and other healthcare providers who attend labor and delivery should give attention for high-risk mothers such grand-multiparty, multiple gustation, and previous history of bleeding to prevent blood loss during childbirth. Finally, birth attendants focus on avoiding unnecessary instrumental delivery, to prevent, and reduce anemia related mortality and morbidity among postpartum women in Ethiopia.

### Electronic supplementary material

Below is the link to the electronic supplementary material.

Supplementary Material 1

## Data Availability

All data included in this systematic review and meta-analysis is available in the manuscript, as additional supporting information, and from the correspondence of this review with reasonable request.

## Abbreviations

ANC: Antenatal Care
APH: Antepartum Hemorrhage
CS: Cesarean Section
EDHS: Ethiopian Demographic Health Survey
IPPA: Immediate postpartum Anemia
MUAC: Mid Upper Arm Circumference
OR: Odds Ratio
PPA: Postpartum Anemia
PPH: Postpartum Hemorrhage
WHO: World Health Organization
